# Dataset on the assessment of the environmental, economic and energy parameters of 5 MW CHP co-gasification plant using South African coal, biomass and waste-tyre

**DOI:** 10.1016/j.dib.2018.10.117

**Published:** 2018-10-27

**Authors:** M. Ozonoh, T.C. Aniokete, B.O. Oboirien, M.O. Daramola

**Affiliations:** a School of Chemical and Metallurgical Engineering, Faculty of Engineering and the Built Environment, University of the Witwatersrand, Wits 2050, Johannesburg, South Africa; b Department of Chemical Engineering, University of Johannesburg, Doornfontein, Johannesburg 2028, South Africa

**Keywords:** Co-gasification, Biomass waste, Coal, Electricity and heat production, Techno-economic, South Africa

## Abstract

The data provided in this article supplements the data information provided in “Techno-economic analysis of electricity and heat production by co-gasification of coal, biomass and waste tyre in South Africa” [1]. The generation of the data considered co-generation of a coal sample (Matla coal) with pine sawdust, sugarcane bagasse, corn cob, and waste tyre at a blend ratio of 1:1, 3:2, and 4:1. The cost evaluation of the use of the feedstocks was considered with feedstock costing (WFC) and without feedstock costing (WOFC). Profitability assessment tools for the case study included NPV, IRR and PBP. The data as contained in this article could be useful for a quick decision making on a similar project by the government and stakeholders in the sector.

**Specifications table**

**Table:** 

Subject area	Electricity and Thermal Power Production
More specific subject area	Chemical Engineering
Type of data	Table, graph, figure
How data was acquired	CHNS-O Organic Elemental Analyzer (FLASH 2000), TGA-SDT (600), Oxygen-bomb calorimeter (203 M 1241).
Data format	Raw and analyzed.
Experimental factors	Feedstocks of South African origin were milled (size reduction) and sieved for analysis.
Experimental features	Results from the proximate and ultimate analysis was fitted into an empirical model to estimate the LHV of the fuels, and was then used in the model equations shown in the experimental design, material and method section, to determine the annual feed rate and feedstock requirements for the 5 MW electric and thermal power plant.
Data source location	Johannesburg, South Africa
Data accessibility	Data are with this article
Related research article	Techno-economic analysis of electricity and heat production by co-gasification of coal, biomass and waste tyre in South Africa [1]

**Value of the data**

• South African coal reserve depletes very fast, and the CO_2_ emission in the country is the highest in the whole of Africa. In this regard, the use of this data article could be instrumental to the reduction in the rapid depletion of the coal reserve, and emissions.
• As for as could be ascertained, data describing the electrical and thermal power production in a 5 MW CHP plant using South African feedstock is not available in the open literature; thus, a set of data provided in this article, could be used as a platform for decision making and further R&D in this area.
• With the provided dataset, investors can have a good understanding of the techno-economic analysis of the power generation in the plant before embarking on the investment, meaning that the dataset provides a working guide for interested investors.
• Policy-making in energy, economic and environmental sectors could consider the dataset, for the modification of existing policies.

## 1 Data

**Fig. 1:**
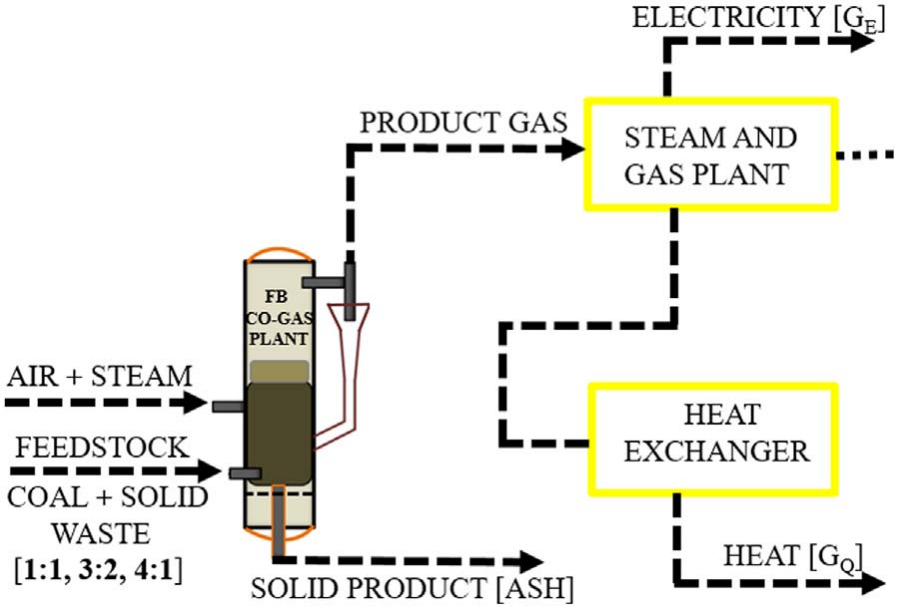
Fluidised bed co-gasification CHP plant [5 MW]: proposed technical approach.

**Fig. 2:**
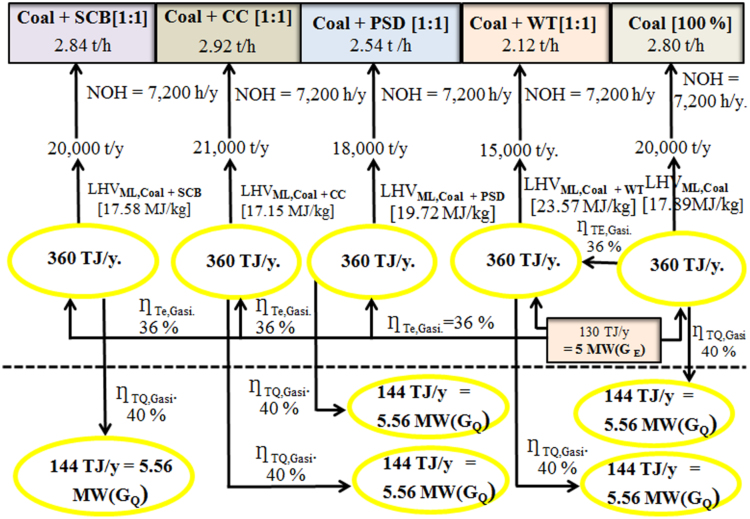
Proposed process flow diagram for the fluidized bed co-gasification CHP plant (5 MW).

**Fig. 3:**
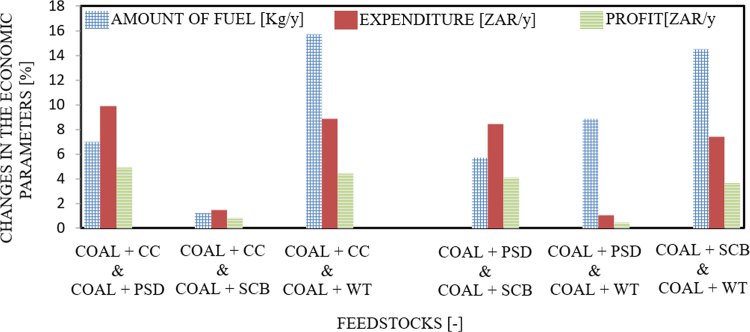
Comparism of economic parameters of the feedstocks at WFC.

**Fig. 4:**
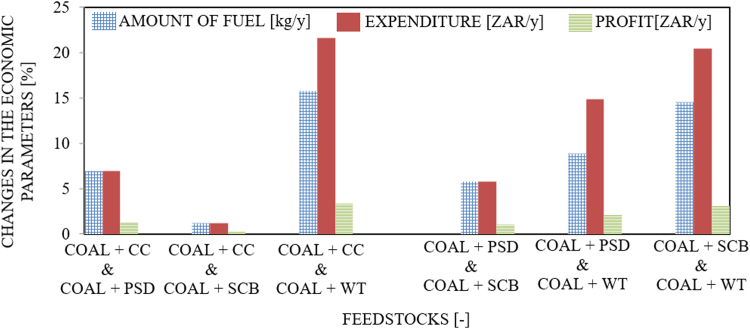
Comparism of economic parameters of the feedstocks at WOFC.

**Fig. 5:**
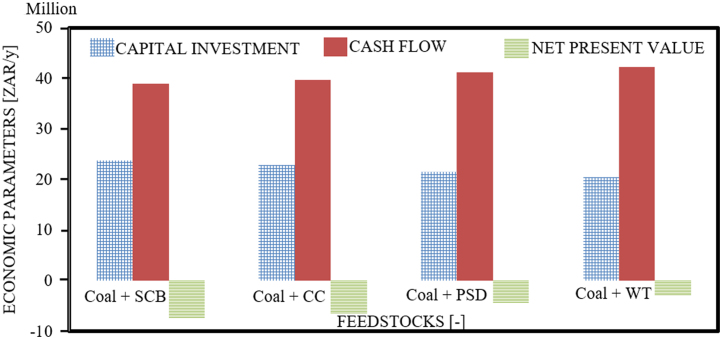
Economic assessment at 18th year at WFC with blend ratio of 3:2.

**Fig. 6:**
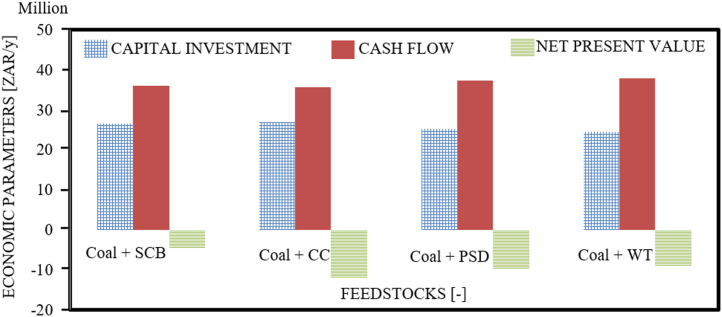
Economic assessment at 18th year at WFC with blend ratio of 4:1.

The dataset provided in this article supplements the data information provided in [1], recently published in the Journal of Cleaner Production, and it contains 11 tables and 6 figures. Fig. 1, Fig. 2 present the flowchart of the proposed technical approach and the scheme of the 5 MW co-gasification process plant. Tables 1 and 2 are the variation in feedstocks economic parameters at the 10th year estimated with feedstock costing (WFC) and without feedstock costing (WOFC), respectively, and both data were estimated using the lower heating value (LHV) data, moisture content (MC) data and the South Africa and other parts of the globe feedstock prices [1]. Tables 3 and 4 emphasize the results of the appraisal tools at the 10th year assessment at WFC and WOFC. Tables 5 and 6 indicate the business viability estimation at the 11th year, at WFC and WOFC. The economic evaluation at the 17th year at WOFC is shown in Table 7. Fig. 3, Fig. 4, Fig. 5, Fig. 6 depict the comparism of the feedstocks economic parameters obtained at WFC and WOFC, as well as the economic assessment at the 18th year with blend ratio 3:1 and 4:1. Table 8 describes the emissions reduction assessment of the plant via co-gasification, while Table 9 presents the statistical sensitivity analysis of the statistical estimation showing the mean of the variables amount of feedstock, capital cost investment, cash flows and net present value (NPV), as well as the variance, standard deviation and standard error of the overall evaluations at 10th year, using WOFC.

**Table 1:** Variation in feedstocks economic parameters ^*“*^[10th year]: WFC.

Feedstocks	Amount of Fuel [t/y]	Expenditure [ZAR/y]	Profit [ZAR/y]	Feedstock with Highest Profit	Percentage Profit [%]
WFC					
Coal+CC &	20,986.05	22,900,607.05	39,739,393.16	Coal+SCB	4.93
Coal+SCB	20,473.45	22,238,058.01	4,0401,942.41		
VEP^*^	5125.49	662,549.42	662,549.22		
Coal+CC &	20,986.05	22,900,607.19	39,739,393.16	Coal+PSD	0.83
Coal+PSD	18,251.81	18,775,998.00	43,864,002.00		
VEP^**^	2734.24	4,124,609.47	4,124,609.03		
Coal+CC &	20,986.05	22,900,607.11	39,739,393.15	Coal+WT	4.48
Coal+WT	15,276.28	19,175,395.25	43,464,605.06		
VEP^***^	5709.77	3,725,212.03	3,725,212.00		
Coal+PSD &	18,251.81	18,775,998.04	43,864,002.42	Coal+PSD	4.11
Coal+SCB	20,473.45	22,238,058.15	40,401,942.15		
VEP^**#**^	2221.65	3,462,060.11	3,462,060.42		
Coal+PSD &	18,251.81	18,775,998.42	43,864,002.11	Coal+PSD	0.46
Coal+WT	15,276.28	19,175,395.02	43,464,605.02		
VEP^**##**^	2975.53	399,397.00	399,397.15		
Coal+SCB &	20,473.45	22,238,058.31	40,401,942.33	Coal+WT	3.65
Coal+WT	15,276.28	19,175,395.08	43,464,605.12		
VEP^**###**^	5197.17	3,062,663.04	3,062,663.04		

*Coal+CC & Coal+SCB; **: Coal+CC & Coal+PSD; ***: Coal+CC & Coal+WT; #: Coal+PSD & Coal+SCB; ##: Coal+PSD & Coal+WT; ###: Coal+SCB & Coal+WT; +: Kg/Yr; ++: ZAR/Yr; VEP: variation in the economic parameters; “: blending ratio of 1: 1; WFC: with feedstock costing.

**Table 2:** Variation in feedstocks economic parameters ^*“*^[10th year]: WOFC.

Parameters	Amount of fuel [t/y]	Expenditure [ZAR/y]	Profit [ZAR/y]	Feedstock with highest profit	Percentage profit [%]
WOFC [except coal]					
Coal+CC &	20,986.05	10,283,164.00	5,356,836.06	Coal+SCB	1.26
Coal+SCB	20,473.45	10,031,991.21	52,608,009.35		
VEP^*^	5125.99	251,173.35	251,173.03		
Coal+CC &	20,986.05	10,283,165.00	52,356,836.12	Coal+PSD	0.24
Coal+PSD	18,251.81	8,943,385.03	53,696,615.00		
VEP^**^	2734.24	1,339,779.11	1,339,779.07		
Coal+CC &	20,986.05	10,283,164.42	52,356,836.44	Coal+WT	3.37
Coal+WT	15,276.26	6,629,905.06	56,010,095.21		
VEP^***^	5709.77	3,402,087.14	3,653,260.07		
Coal+PSD &	18,251.81	8,943,385.11	53,696,615.14	Coal+PSD	1.02
Coal+SCB	20,473.45	10,031,991.00	52,608,009.25		
VEP^**#**^	2221.65	1,088,606.02	1,088,606.36		
Coal+PSD &	18,251.81	894,338.14	53,696,615.11	Coal+WT	2.11
Coal+WT	15,276.28	6,629,905.25	56,010,095.07		
VEP^**##**^	2975.53	2,313,481.11	2,313,481.04		
Coal+SCB &	20,473.45	10,031,991.04	52,608,009.16	Coal+WT	3.13
Coal+WT	15,276.28	6,629,905.06	56,010,095.22		
VEP^**###**^	5197.17	3,402,087.17	3,402,087.00		

*: Coal+CC & Coal+SCB; **: Coal+CC & Coal+PSD; ***: Coal+CC & Coal+WT; #: Coal+PSD & Coal+SCB; ##: Coal+PSD & Coal+WT; ###: Coal+SCB & Coal+WT; ^**“**^: with a blending ratio of: 1:1; WOFC: without feedstock costing; VEP: variation in the economic parameter.

**Table 3:** Plant assessment at 10th year, WFC: emphasis on appraisal tools.

Feedstocks [-]	Amount of fuel [t]	Capital cost investment [δ] [ZAR/y]	Cash flow [μ] [ZAR/y]	Net present value [NPV] [ZAR/y]	Internal rate of return [IRR] (%)	Payback period [PBP] (Year)
Ratio: [1:1], Interest rate: [5%] - WFC						
Coal+SCB	20,473.45	22,238,058.18	40,401,941.82	25,652,29.37	6.15	0.55
Coal+CC	20,986.05	22,900,607.30	39,739,392.70	1,495,932.57	5.66	0.58
Coal+PSD	18,251.81	18,775,980.37	43,864,001.63	8,152,693.59	8.85	0.43
Coal+WT	15,276.28	19,175,395.01	43,464,604.99	7,508,102.06	8.52	0.44

Ratio: [3:2], Interest rate: [5%] - WFC						
Coal+SCB	20,401.90	23,695,579.21	38,944,420.79	212,916.87	5.09	0.61
Coal+CC	20,807.06	22,918,288.46	39,721,711.54	1,467,396.71	5.65	0.58
Coal+PSD	19,270.94	21,496,041.75	41,143,958.25	3,762,779.53	6.70	0.52
Coal+WT	15,743.76	20,414,617.14	42,225,382.86	5,508,105.04	7.73	0.48

Ratio: [4:1], Interest rate: [5%] - WFC						
Coal+SCB	20,260.28	26,580,269.34	36,059,730.66	−4,442,722.76	3.09	0.74
Coal+CC	20,458.08	26,881,014.28	35,758,985.72	−4,928,099.02	2.89	0.75
Coal+PSD	19,686.61	25,237,300.14	37,402,699.86	−2,275,286.98	4.01	0.67
Coal+WT	17,855.69	24,632,996.13	38,007,003.87	−1,299,992.72	4.43	0.65

**Table 4:** Plant assessment at 10th year, WOFC: emphasis on appraisal tools.

Feedstocks [-]	Amount of fuel [t]	Capital cost investment [δ] [ZAR/y]	Cash flow [μ] [ZAR/y]	Net present value [NPV] [ZAR/y]	Internal rate of return [IRR] (%)	Payback period [PBP] (Year)
Ratio: [1:1], Interest rate: [5%] - WFC						
Coal+SCB	20,473.45	10,031,991.19	52,608,008.81	22,264,762.66	18.02	0.17
Coal+CC	20,986.05	10,283,164.48	52,356,835.52	21,859,390.76	17.67	0.17
Coal+PSD	18,251.81	8,943,385.18	53,696,614.82	24,021,678.33	19.63	0.16
Coal+WT	15,276.28	6,629,904.59	56,010,095.41	27,755,435.32	23.78	0.14

Ratio: [3:2], Interest rate: [5%] - WFC						
Coal+SCB	20,401.90	9,711,302.96	52,928,697.04	22,782,325.65	18.47	0.18
Coal+CC	20,807.06	9,904,159.23	52,735,840.77	22,471,072.36	18.20	0.19
Coal+PSD	19,270.94	9,172,968.27	53,467,031.74	23,651,151.15	19.27	0.17
Coal+WT	15,743.76	6,788,708.79	55,851,291.21	27,499,139.12	23.46	0.12

Ratio: [4:1], Interest rate: [5%] - WFC						
Coal+SCB	20,260.28	9,076,604.62	53,563,395.38	23,806,673.71	19.43	0.10
Coal+CC	20,458.08	9,165,218.93	53,474,781.07	23,663,657.91	19.29	0.20
Coal+PSD	19,686.61	8,819,602.70	53,820,397.30	24,221,452.51	19.83	0.17
Coal+WT	17,855.69	7,599,381.80	55,040,618.20	26,190,783.20	21.90	0.12

WOFC: without feedstock costing.

**Table 5:** Estimation of the business viability at the 11th year: WFC.

Feedstocks [-]	Capital cost investment [δ] [ZAR/y]	Cash flow [μ] [ZAR/y]	Net present value [NPV] [ZAR/y]	Internal rate of return [IRR] (%)	Payback period [PBP] (Year)
Ratio: [1:1], Interest rate: [5%] - WFC					
Coal+SCB	22,238,058.18	40,401,941.82	1,384,120.43	5.58	0.6
Coal+CC	22,900,607.30	39,739,392.70	334,192.58	5.13	0.6
Coal+PSD	18,775,98O.37	43,864,001.63	6,870,374.92	8.01	0.5
Coal+WT	19,175,395.01	43,464,604.99	6,237,459.34	7.71	0.5

Ratio: [3:2], Interest rate: [5%] - WFC					
Coal+SCB	23,695,579.21	38,944,420.79	−925,582.96	4.62	0.6
Coal+CC	22,918,288.46	39,721,711.54	306,173.61	51.2	0.6
Coal+PSD	214,96,041.75	41,143,958.25	2,559,978.51	6.08	0.4
Coal+WT	20,414,617.14	42,225,382.86	4,273,689.70	6.83	0.4

Ratio: [4:1], Interest rate: [5%] - WFC					
Coal+SCB	26,580,269.34	36,059,730.66	−4,442,722.76	2.81	0.7
Coal+CC	26,881,014.28	35,758,985.72	−5,973,475.94	2.63	0.8
Coal+PSD	25,237,300.14	37,402,699.86	−3,368,716.17	3.64	0.7
Coal+WT	24,632,996.13	38,007,003.87	−2,411,088.12	4.02	0.7

WFC: with feedstock costing.

**Table 6:** Estimation of the business viability at the 11th year: WOFC.

Feedstocks [-]	Capital cost investment [δ] [ZAR/y]	Cash flow [μ] [ZAR/y]	Net present value [NPV] [ZAR/y]	Internal rate of return [IRR] [%]	Payback period [PBP] [Year]
Ratio: [1:1], Interest rate: [5%] - WOFC					
Coal+SCB	10,031,991.19	52,608,008.81	20,726,822.00	16.23	0.2
Coal+CC	10,283,164.48	52,356,835.52	20,328,792.89	15.95	0.2
Coal+PSD	8,943,385.18	53,696,614.82	22,451,913.40	17.69	0.2
Coal+WT	6,629,904.59	56,010,095.41	26,118,038.18	21.41	0.1

Ratio: [3:2], Interest rate: [5%] - WOFC					
Coal+SCB	9,711,302.96	52,928,697.04	21,235,010.00	18.47	0.2
Coal+CC	9,904,159.23	52,735,840.77	20,929,394.67	16.42	0.2
Coal+PSD	9,172,968.27	53,467,031.74	22,088,097.84	17.38	0.2
Coal+WT	6,788,708.79	55,851,291.21	25,866,384.46	21.12	0.1

Ratio: [4:1], Interest rate: [5%] - WOFC					
Coal+SCB	9,076,604.62	53,563,395.38	22,240,803.31	17.51	0.2
Coal+CC	9,165,218.93	53,474,781.07	22,100,378.06	17.39	0.2
Coal+PSD	8,819,602.70	53,820,397.30	22,648,068.92	17.87	0.2
Coal+WT	7,599,381.80	55,040,618.20	24,581,727.73	19.73	0.1

WOFC: without feedstock costing.

**Table 7:** Economic evaluation at the 17th Year: WOFC.

Feedstocks [-]	Capital cost investment [δ] [ZAR/y]	Cash flow [μ] [ZAR/t]	Net present value [NPV] [ZAR/y]	Internal rate of return [IRR] [%]	Payback period [PBP] [Year]
Ratio: [1:1], Interest rate: [5%] - WOFC					
Coal+SCB	22,238,058.18	40,401,941.82	12,920,708.80	26.22	0.2
Coal+CC	22,900,607.30	39,739,392.70	12,559,949.43	26.00	0.2
Coal+PSD	18,775,998.37	43,864,001.63	14,484,270.01	27.24	0.2
Coal+WT	19,175,395.01	43,464,604.99	17,807,114.51	29.82	0.1

Ratio: [3:2], Interest rate: [5%] - WOFC					
Coal+SCB	23,695,579.21	38,944,420.79	13,381,312.24	18.47	0.2
Coal+CC	22,918,288.46	397,21,711.54	13,104,313.42	11.01	0.2
Coal+PSD	21,496,041.75	41,143,958.25	1,4154,520.58	10.34	0.2
Coal+WT	20,414,617.14	42,225,382.86	17,579,024.57	10.92	0.1

Ratio: [4:1], Interest rate: [5%] - WOFC					
Coal+SCB	26,580,269.34	36,059,730.66	14,292,927.36	11.01	0.2
Coal+CC	26,881,014.28	35,758,985.72	14,165,650.93	10.93	0.2
Coal+PSD	252,37,300.14	37,402,699.86	14,662,058.36	11.22	0.2
Coal+WT	24,632,996.13	38,007,003.87	16,414,657.61	12.36	0.1

WOFC: without feedstock costing.

**Table 8:** Emission reduction assessment from the plant (Coal & Coal+Solid Waste).

Feedstock	CO [kg]	CO_2_ [kg]	SO_2_ [kg]	NO_X_ [kg]
Coal **(Matla)**	1.00	5900.00	69.50	26.00

Blending ratio: [1:1]				
Coal+SCB	0.50	2950.01	34.75	13.00
Coal+CC	0.50	2950.01	34.75	13.00
Coal+PSD	0.50	2950.01	34.75	13.00
Coal+WT	0.50	2950.01	34.75	13.00

Blending ratio: [3:2]				
Coal+SCB	0.60	3540.42	41.70	15.60
Coal+CC	0.60	3540.42	41.70	15.60
Coal+PSD	0.60	3540.42	41.70	15.60
Coal+WT	0.60	3540.42	41.70	15.60

Blending ratio: [4:1]				
Coal+SCB	0.80	4720.00	55.60	20.80
Coal+CC	0.80	4720.00	55.60	20.80
Coal+PSD	0.80	4720.00	55.60	20.80
Coal+WT	0.80	4720.00	55.60	20.80

SCB: sugarcane bagasse; CC: corn cob; PSD: pine saw-dust; WT: waste tyre.

**Table 9:** Sensitivity analysis WOFC: At 10th year (1:1, 3:2, 4:1 Coal+Solid Waste).

Amount of feedstock [t]			Capital cost investment [ZAR/y]			Ratio
X	$\bar{X}$	${\left(X-\bar{X}\right)}^{2}$	X	$\bar{X}$	${\left(X-\bar{X}\right)}^{2}$	
20,473.45*	18,746.90	2.98099E+9	10,031,991.19	16,547,914.72	4.24573E+13	**1:1**
20,986.05**	18,746.90	5.01381E+9	10,283,164.48	16,547,914.72	3.92471E+13	
18,251.81***	18,746.90	2.45114E+8	8,943,385.18	16,547,914.72	5.78289E+13	
15,276.28****	18,746.90	1.20452E+10	6,629,904.59	16,547,914.72	9.83669E+13	
Variance	6.7617E+9		Variance	2.77632E+12		
Std. deviation	2600.33		Std. deviation	1,666,229.60		
Standard Error	1300.16		Standard Error	833,114.80		
Cash flow [ZAR/y]			Net present value [ZAR/y]			
52,608,008.81	41,867,485.29	1.15359E+14	22,264,762.66	3,706,536.82	3.44408E+14	
52,356,835.52	41,867,485.29	1.10026E+14	21,859,390.76	3,706,536.82	3.29526E+14	
53,696,614.82	41,867,485.29	1.39928E+14	24,021,678.33	3,706536.82	4.12705E+14	
56,010,095.41	41,867,485.29	2.00013E+14	27,755,435.32	3,706,536.82	5.7835E+14	
Variance	2.77632E+12		Variance	7.23153E+12		
Std. deviation	1,666,229.60		Std. deviation	2,689,150.05		
Standard Error	833,114.80		Standard Error	1,344,575.02		
Amount of feedstock [t]			Capital cost investment [ZAR/y]			
20,401.90*	19,055.91	1.81167E+9	9,711,302.96	22,131,131.64	1.54252E+14	**3:2**
20,807.06**	19,055.91	3.0665E+9	9,904,159.23	22,131,131.64	1.49499E+14	
19,270.94***	19,055.91	4.623706E+7	9,172,968.27	22,131,131.64	1.67914E+14	
15,743.76****	19,055.91	1.09704E+10	6,788,708.79	22,131,131.64	2.3539E+14	
Variance	5.29826E+9		Variance	2.06616E+12		
Std. Deviation	2301.80		Std. deviation	1,437,414.328		
Standard Error	1150.90		Standard Error	718,707.1639		
Cash flow [ZAR/y]			Net present value [ZAR/y]			
52,928,697.04	40,508,868.36	1.54252E+14	22,782,325.65	2,737,799.53	4.01783E+14	
52,735,840.77	40,508,868.36	1.49499E+14	22,471,072.36	2,737,799.53	3.89402E+14	
53,467,031.74	40,508,868.36	1.67914E+14	23,651,151.15	2,737,799.53	4.37368E+14	
55,851,291.21	40,508,868.36	2.3539E+14	27,499,139.12	2,737,799.533	6.13124E+14	
Variance	2.06616E+12		Variance	5.38176E+12		
Std. deviation	1,437,414.33		Std. deviation	2,319,862.04		
Standard Error	718,707.16		Standard Error	1,159,931.02		
Amount of feedstock [t]			Capital cost investment [ZAR/y			
20,260.28^*^	19,565.16	4.83182E+11	9,076,604.62	25,832,894.97	2.80773E+14	
20,458.08^**^	19,565.16	7.97294E+11	9,165,218.93	25,832,894.97	2.77811E+14	
19,686.61^***^	19,565.16	1.474969E+10	8,819,602.70	25,832,894.97	2.89452E+14	
17,855.69^****^	19,565.16	2.9223E+12	7,599,381.80	25,832,894.97	3.32461E+14	
Variance	1.40584E+12		Variance	5.2636E+11		
Std. deviation	1,185,682.54		Std. deviation	725,507.02		
Standard Error	592,841.27		Standard Error	362,753.51		
Cash flow [ZAR/y]			Net present value [ZAR/y]			
53,563,395.38	36,807,105.03	2.80773E+14	23,806,673.71	−3,236,525.37	7.31335E+14	**4:1**
53,474,781.07	36,807,105.03	2.77811E+14	23,663,657.91	−3,236,525.37	7.2362E+14	
53,820,397.30	36,807,105.03	2.89452E+14	24,221,452.51	−3,236,525.37	7.53941E+14	
55,040,618.20	36,807,105.03	3.32461E+14	26,190,783.26	−3,236,525.37	8.65966E+14	
Variance	5.2636E+11		Variance	1.37102E+12		
Std. deviation	725,507.02		Std. deviation	1,170,905.43		
Standard Error	362,753.51		Standard Error	585,452.71		

*: Coal + SCB, **: Coal + CC, ***: Coal + PSD, ****: Coal +WT, X: Estimated variable. X: estimated variable (e.g. amount of feedstock, capital cost investment, cash flow, net present value) $\bar{X}$: mean of the variable; Std.: standard.

## 2 Experimental design, materials and methods

South African feedstocks comprising coal and solid waste (sugarcane bagasse, corn cob, pine saw-dust, and waste-tyre) were pre-treated by milling and sieving and kept in air-tight bags for analysis. Characterization of the feedstocks was carried out using CHNS-O Organic Elemental Analyzer (FLASH 2000), TGA-SDT (600), Oxygen-bomb calorimeter (203 M 1241) for the ultimate, and proximate analysis and heating value determinations, respectively, and using coal-to-solid waste ratios of 1:1, 3:2, and 4:1. The result from characterization analysis was used in the empirical model (Eq. (1)) to obtain the lower heating value (LHV) of the fuels, and the value was then applied in Eqs. (2) and (3) to estimate the annual feed rate and feedstock requirements shown in the model for the co-gasification power plant presented in Fig. 2

(1) $$\mathrm{LHV}=\mathrm{HHV}-\left(0.212\times M_{H}\right)-\left(0.0245\times \mathrm{MC}\right)$$

(2) $$F_{\mathrm{R ANNUAL}}=\frac{\gamma }{\frac{{\mathrm{LHV}}_{\mathrm{FEEDSTOCK}}}{\mathrm{NOH}}}$$

(3) $$A_{\mathrm{FR}}=\frac{\varpi \times 3.6}{\mathrm{LHV}\times \eta_{0}}$$

where LHV and HHV are the lower heating value and higher heating value of the feedstocks in MJ/kg, respectively; $F_{\mathrm{R ANNUAL}}$ is the annual feed-rate of the plant in t/y; $A_{\mathrm{FR}}$ is the annual feedstock requirement of the plant in t/y; $\eta_{0}$ is the operating efficiency of the plant in %, respectively, $M_{H}$ and MC are the mass fractions of hydrogen and moisture content of the feedstocks in %, NOH is the number of operating hours in the plant in h; ϖ is the energy demand in MWh/y; and γ is the gasification conversion efficiency in %.

The economic evaluation of electricity and thermal power generation from the plant was then carried out using the net present value (NPV), internal rate of return (IRR), and payback period (PBP) as the project tools at 10th, 11th, 17th and 18th investment years based on two financial situations namely: with feedstock costing (WFC) and without feedstock costing (WOFC). The appraisal tools and emission models used are provided in Eqs. (4)–(10). The NPV was estimated with Eq. (4) [2]. Eqs. (5) and (6) [2] were applied to estimate the IRR and PBP, respectively. Eq. (7) [3] and Eq. (8) [4] were used to estimate the emissions from the plant. Sensitivity analysis that considers the standard deviation, variance, and standard error was carried out on the variables using Eqs. (9), (10), and (11), respectively [5]

(4) $$\mathrm{NPV}=-\beta \frac{\phi_{1}}{{\left(1+R\right)}^{1}}+\frac{\phi_{2}}{{\left(1+R\right)}^{2}}+\frac{\phi_{3}}{{\left(1+R\right)}^{3}}+..\ldots \ldots \ldots \frac{\phi }{{\left(1+R\right)}^{T}}$$

(5) $$\mathrm{NPV}=-\beta +\sum_{j=1}^{T}\frac{\phi_{j}}{{\left(1+\mathrm{IRR}\right)}^{j}}=0$$

(6) $$\mathrm{PBP}=\frac{\delta }{\mu }$$

where NPV, IRR, and PBP are the net present value, internal rate of return, and payback period, respectively; β is the capital investment in ZAR/y; ϕ is the cash flow in million (M) ZAR; R is the annual rate of return in %; T is the economic life of the plant or business period

(7) $$\xi =\varpi \times \left[(\alpha^{1}\times \tau^{1})+(\alpha^{2}\times \tau^{2})\right]+\cdots \cdots \cdots \cdots \alpha^{n}\times \tau^{m}$$

(8) φ=ξ−ε−λ

where ξ is the emission reduction by displaced energy from the grid; φ is the effective emission reduction; ε is the life cycle GHG emission intensity of biomass; λ is the emission from transportation of biomass; α is the percentage of feedstock used for the blend; τ is the emission factor of the fuel used

(9) $$\mathrm{SD}=\sqrt{\frac{\Sigma (Χ-{\bar{Χ})}^{2}}{N-1}}$$

(10) $$\mathrm{Variance}=\frac{\Sigma (Χ-{\bar{Χ})}^{2}}{N-1}$$

(11) $$\mathrm{Standard}\;\mathrm{Error}=\frac{\mathrm{SD}}{{\left(N\right)}^{1/2}}$$

SD is the standard deviation of the sensitivity analysis variables; Χ is the variables including the amount of feedstock, capital cost investment, cash flow, and NPV; N is the number of sample population.
